# Development and Application of a Novel Pluri-Residue Method to Determine Polar Pesticides in Fruits and Vegetables through Liquid Chromatography High Resolution Mass Spectrometry

**DOI:** 10.3390/foods9050553

**Published:** 2020-05-01

**Authors:** Lorena Manzano-Sánchez, José Antonio Martínez-Martínez, Irene Domínguez, José Luis Martínez Vidal, Antonia Garrido Frenich, Roberto Romero-González

**Affiliations:** Department of Chemistry and Physics, Analytical Chemistry Area, University of Almeria, Center for Research in Mediterranean Intensive Agrosystems and Agri-Food Biotechnology (CIAIMBITAL), Agrifood Campus of International Excellence ceiA3, Carretera de Sacramento s/n, E-04120 Almeria, Spain; lorenamanzano@ual.es (L.M.-S.); joseantonio93martinez@gmail.com (J.A.M.-M.); idominguez@ual.es (I.D.); jlmartin@ual.es (J.L.M.V.); agarrido@ual.es (A.G.F.)

**Keywords:** high polar pesticides, UHPLC-Orbitrap-MS, QuPPe, pluri-residue analysis

## Abstract

Nowadays, highly polar pesticides are not included in multiresidue methods due to their physico-chemical characteristics and therefore, specific analytical methodologies are required for their analysis. Laboratories are still looking for a pluri-residue method that encompasses the largest number of polar pesticides. The aim of this work was the simultaneous determination of ethephon, 2-hydroxyethylphosphonic acid (HEPA), fosetyl aluminum, glyphosate, aminomethylphosphonic acid (AMPA), N-acetyl-glyphosate and N-acetyl-AMPA in tomatoes, oranges, aubergines and grapes. For that purpose, an ultra high performance liquid chromatography (UHPLC) coupled to a high resolution single mass spectrometer Orbitrap-MS were used. Different stationary phases were evaluated for chromatographic separation, and among them, the stationary phase Torus DEA provided the best separation of the selected compounds. The QuPPe method was used for the extraction of the analytes, but slight modifications were needed depending on the matrix. The developed method was validated, observing matrix effect in all matrices. Intra- and inter-day precision were estimated, and relative standard deviation were lower than 19%. Recoveries were satisfactory, and mean values ranged from 70% to 110%. Limits of quantification were between 25 and 100 µg kg^−1^. Finally, the analytical method was applied to different fruits and vegetables (oranges, tomatoes, aubergines and grapes).

## 1. Introduction

High polar pesticides have different physico-chemical characteristics compared with other pesticides, and therefore, they are not included in multiresidue methods, and a pluri-residue method is required for their simultaneous analysis [1]. The main problems are unsuitable extraction, due to low or null affinity for the organic phase, and the incompatibility with conventional reverse phases because these compounds are poorly retained, and therefore, bad peak shapes are obtained. In order to sort out these analytical problems, ion pairing agents [2,3] or derivatization processes [4,5,6] are used. However, in the last few years, the most common strategy is based on using new stationary phases, due to the fact that derivatization increases the manipulation of the sample, increasing errors associated to this step.

The European Reference Laboratory (EURL, EU Reference Laboratory) developed a generic method, named Quick Polar Pesticides Methods (QuPPe) [7], based on the extraction of polar pesticides from the sample with acidified methanol and liquid chromatography coupled to tandem mass spectrometry (LC-MS/MS). This was used for the determination of polar pesticides in different food [8] or biological matrices [9]. The QuPPE method proposes the use of different stationary phases such as graphitized porous carbon (Hypercarb), hydrophilic interaction liquid chromatography (HILIC) or ionic exchange. However, the authors themselves [7] pointed out numerous issues that require additional studies, such as: (i) interaction of the analytes with the active sites of the column and deterioration of the stationary phase; (ii) similar *m/z* transitions of several compounds; (iii) degradation of fosetyl-Al and ethephon to phosphonic acid and (iv) possible “matrix effects” that could affect the quality of the results, among others. 

The QuPPe method was modified by several authors. Some of them used additional cleaning steps with OASIS cartridges [10,11] or carbon nanotubes [12]. Other authors utilized alternative methods, such as extraction with acetonitrile and *n*-hexane for the extraction of ethephon from tomatoes [13], or the use of an aqueous solution of ethylenediaminetetraacetic acid (EDTA) acidified with acetic acid to improve the extraction of glyphosate, glufosinate and AMPA from grapes [11].

In relation to chromatographic separation, the current bibliography provides various alternatives. For the determination of glyphosate, glufosinate and their metabolites, Hypercarb [14,15] or HILIC [16,17] stationary phases were used. Alternatively, for glyphosate, ethephon and fosetyl-Al, mixed mode columns were tested, such as Acclaim Trinity Q1 [7], due to its versatile retention mechanism, and Obelisc N [18], obtaining better retention time reproducibility and robustness than those obtained with HILIC stationary phases. However, for the simultaneous determination of different polar pesticides, including glyphosate and glufosinate metabolites among others, HILIC provided the best results, while for glyphosate and AMPA, the mixed-mode separation column Obelisc N [16] offered the best values in terms of retention times and peak shape reproducibility. Other authors eliminated the chromatographic separation stage for the simultaneous analysis of different polar pesticides (ethephon, fosetyl-Al, glyphosate, glufosinate and metabolites), although a strong matrix effect was observed and this required a high dilution of the extract, and therefore, analytical sensitivity was affected [19]. In recent years, the use of supercritical fluid chromatography has also been proposed for the simultaneous separation of compounds with a wide polarity range, including quats and fosetyl-Al, using typical reverse phase columns [20]. Other approaches such as the use of parallel columns (HILIC and C18) [21] or ion chromatography [8,22,23] were also tested, as well as the use of isotopic labelled internal standards for each analyte, but this approach increases the cost of the analysis [24].

The aim of this study was the development of a pluri-residue method for the simultaneous determination of polar pesticides (ethephon, fosetyl, glyphosate) and metabolites (2-hydroxyethylphosphonic acid (HEPA), aminomethylphosphonic acid (AMPA), n-acetyl-AMPA, n-acetyl-glyphosate) in different matrices (fruits and vegetables), testing different stationary phases as well as introducing some modifications to the QuPPe method. For the detection of the compounds, a high resolution mass spectrometry (HRMS) analyzer was used bearing in mind that high mass accuracy monitorization of the characteristic ion and fragments can be performed [15,25], increasing the reliability of the identification process.

## 2. Materials and Methods

### 2.1. Reagents and chemicals

Ethephon, HEPA, fosetyl-Al, glyphosate, N-acetyl-glyphosate, AMPA, and N-acetyl-AMPA reference standards were purchased from Dr Ehrenstorfer GmbH (Schlosser, Augsburg, Germany). N-Acetyl-d3-glufosinate, used as the internal standard, was acquired from Sigma-Aldrich (Saint Louis, MO, USA). Purity of all compounds was ≥99.7%.

Stock standard solutions of each compound (1 mg mL^−1^) were prepared by exact weighing of the solid substances and dissolved in 50 mL of solvent (methanol or a mixture of methanol:water), according to the instructions provided by EURL [7], and they were stored at −18 °C without being exposed to light. Then, a working standard solution (at 10 mg L^−1^), containing the polar pesticides, was prepared in an aqueous solution (10% acetonitrile) and was stored as the stock standard solutions. The stock standard solutions were stable up to one year and working standard solutions were prepared every two months.

LC-MS grade methanol, acetonitrile and water were purchased from Honeywell (LC-MS grade, Morrison, NJ, USA) while ultrapure water was obtained by a Milli-Q water gradient system (Millipore, Bedford, MA, USA). Formic acid was purchased from Fisher Scientific (Erembodegem, Belgium).

Finally, 0.22 µm nylon syringe filters were used and they were acquired from Agilent Technologies (Santa Clara, CA, USA).

### 2.2. Apparatus and Instrument

A Reax 2 rotatory shaker from Heidolph (Schwabach, Germany) was used to extract polar pesticides from the samples. WX vortex from Velp Scientifica (Usmate, Italy) and a Polytron PT 2100 from Kinematica (Luzern, Switzerland) were utilized for the homogenization of the samples. To centrifuge the extracts, a Centronic-PL II centrifuge from JP Selecta (Barcelona, Spain) was used.

For the analysis of the targeted compounds, Thermo Fisher Scientific Transcend 600 LC (Thermo Scientific Transcend™, Thermo Fisher Scientific, San Jose, CA, USA) was utilized. LC system was coupled to a high resolution single mass spectrometer Exactive-Orbitrap analyzer (Thermo Fisher Scientific, Bremen, Germany) and ionization was performed using an electrospray interface (ESI) (HESI-II, Thermo Fisher Scientific, San Jose, CA, USA).

The chromatographic separation was carried out with a Torus DEA column (100 × 2.1 mm, 1.7 µm particle size) (Waters, Milford, MA, USA). Moreover, four columns were also tested during the optimization of the method: Obelisc N (100 × 2.1 mm, 5 µm particle size) (Sielc, Wheeling, IL, USA), HILIC-A (250 × 4.6 mm, 3 µm particle size) (ACE, Aberdeen, Scotland), HILIC-B (250 × 4.6 mm, 3 µm particle size) (ACE) and Zorbax HILIC Plus (100 × 2.1 mm, 3.5 µm particle size) (Agilent, Santa Clara, CA, USA).

### 2.3. Samples Collection

Samples were obtained from local supermarkets located in Almeria (Spain). The analyzed samples were tomato (n = 10), orange (n = 10), aubergine (n = 10) and grapes (n = 10). The total number of analyzed samples was 40.

### 2.4. Sample Preparation

Extraction method is based on QuPPe method [7] with some modifications. Briefly, 10 g of sample was weighed in 50-mL polypropylene centrifuge tubes. For orange and aubergine, 1.5 mL and 1 mL of water were added respectively before the addition of 10 mL of acidified methanol (1 % formic acid). The tubes were homogenized with polytron for 1 min, and then, in a rotatory agitator for 10 min. After that, the mixture was centrifuged at 4000 rpm for 5 min. Finally, 1 mL of the supernatant was filtered into a 0.22 µm nylon syringe filter and injected into the LC system.

### 2.5. UHPLC-Orbitrap-MS Analyses

The chromatographic separation was performed using a mobile phase that comprises water (0.9% formic acid) as eluent A, and acidified acetonitrile (0.9% formic acid) as eluent B. The gradient elution started at 10% of A and raised to 90% A in 6 min. This composition was held for 16 min, then decreased to 50 % for 2 min and came back to initial conditions (10% A) for 2 min. Finally, this composition was held for 1 min. Column temperature was kept at 50 °C, injection volume was 10 µL, the flow rate was set at 0.5 mL min^−1^ and the analysis time was 27 min.

The ESI parameters for the spectrometric detection were as follows: spray voltage, 4 kV; sheath gas (N_2_, >95%), 10 (adimensional); capillary voltage, −35 V; skimmer voltage, 18 V; tune lens voltage, 95 V; capillary temperature, 300 °C; heater temperature, 305 °C. Two alternating acquisition functions were used: (1) full MS, ESI^–^, without fragmentation (the higher collisional dissociation (HCD) collision cell was switched off), mass resolving power = 25,000 full width at half maximum (FWHM); scan time = 0.25 s and (2) all-ions fragmentation (AIF), ESI^–^, with fragmentation (HCD on, collision energy 30 eV), mass resolving power = 10,000 FWHM; scan time = 0.10 s. Mass range in the full scan experiment was set at *m/z* 50–500.

The chromatograms and spectra were processed using Xcalibur™ 7.0 (Thermo Fisher Scientific, Les Ulis, France).

### 2.6. Validation

Validation of the optimized method was carried out using the SANTE guidelines [26]. Linearity, matrix effect, limit of quantification (LOQ), trueness, intra- and inter-day precision were evaluated.

To study the linearity of the proposed method, matrix-matched calibration was built, and blank extracted samples were spiked at several concentrations: 25, 50, 100, 50, 1000 µg kg^−1^.

Equation (1) was used to calculate the percentage of matrix enhancement or suppression:

(1) $$\mathrm{Matrix}\;\mathrm{effect}\;\left(\%\right)=\left[\frac{\mathrm{slope}\;\mathrm{in}\;\mathrm{matrix}}{\mathrm{slope}\;\mathrm{in}\;\mathrm{solvent}}-1\right]$$

Matrix effect was considered negligible if it is equal to or lower than ±20%, while values higher than 20% indicate strong matrix enhancement and values lower than −20% indicate considerable matrix suppression.

Indications described in the SANTE guidelines [26] were followed for the estimation of the LOQ, defining this parameter as the lowest concentration of the analyte that has been validated with acceptable trueness (recovery ranging from 70–120%) and precision (RSD lower than 20%). Thus, spiked samples at low concentrations, from 10 to 1000 µg kg^−1^, were extracted and LOQs were estimated in the four matrices evaluated.

Trueness was investigated through recovery studies spiking blank samples at two concentration levels (LOQ and 10 × LOQ), and each concentration level was analyzed five times.

Precision was evaluated by means of repeatability (intra-day precision) and reproducibility (inter-day precision). The results were expressed as relative standard deviation (RSD, %). Five replicates at two concentration levels (LOQ and 10 × LOQ) were evaluated for intra-day precision. For inter-day precision, five replicates at the same concentration levels (LOQ and 10 × LOQ) were tested for 5 days.

## 3. Results and Discussion

### 3.1. Optimization of High Resolution Mass Spectrometry

For the spectrometric characterization of the analytes, a solution of 1 mg L^−1^ in water:acetonitrile (90:10 *v/v*) of each compound was injected into the LC-HRMS system. This analysis was performed in negative ionization mode. The flow rate was 0.2 mL min^−1^ and the mobile phase was composed of a mixture of water:acetonitrile (50:50, *v/v*) both with 0.9% formic acid for 2 min without a chromatographic column due to the fact that separation is not required for this step.

**Figure 1:**
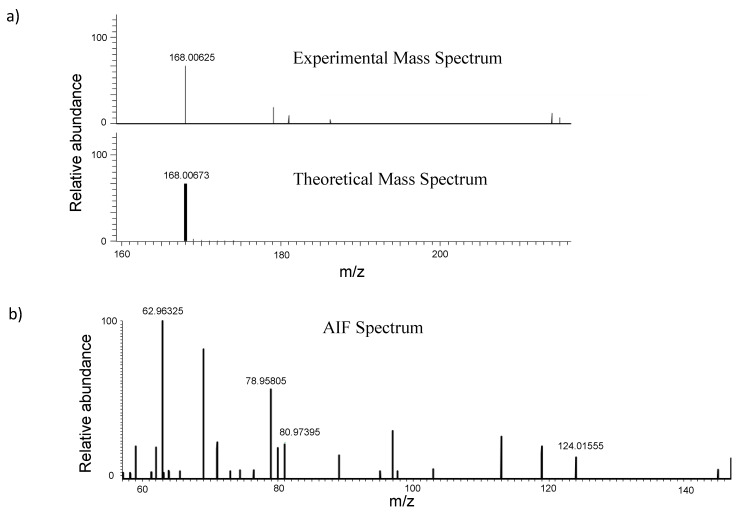
(**a**) Experimental and theoretical MS spectrum of glyphosate. (**b**) Pseudo MS/MS (all ion fragmentation spectrum) of glyphosate.

The exact mass of the characteristic ion was selected in the full scan mass spectrum from molecular formulae. Then, fragments were selected in the pseudo MS/MS spectrum, known as All Ion Fragmentation (AIF). The fragments have to fit the retention time of the characteristic ion, as well as the peak shape should be similar to that obtained by the characteristic ion. Figure 1 shows the high resolution mass spectrum that was obtained for glyphosate.

Spectrometric parameters for all the studied compounds are shown in Table 1, where it can be observed that at least two fragments were monitored for each compound and mass errors were always lower than 5 ppm.

The fragmentation of the different compounds was evaluated and for instance, ethephon’s fragments are due to the loss of a molecule of hydrochloric acid (106.98926 *m/z*) followed by loss of ethene (78.95795 *m/z*). HEPA loses a molecule of water as well as ethene to give the metaphosphate ion (78.95795 *m/z*). In addition, two fragments were monitored for HEPA, corresponding to the loss of formaldehyde and metaphosphoric ion, 94.98926 and 59.01276 *m/z* respectively. For fosetyl aluminum, metaphosphite ion (62.96304 *m/z*) is obtained by loss of ethanol, whereas the ion corresponding to metaphosphate (78.95795 *m/z*) was monitored because the loss of ethane; moreover, the characteristic ion of phosphonic acid (80.97360 *m/z*) is obtained by the loss of an ethene molecule.

A similar fragmentation pathway was obtained for these compounds because they belong to the same family of organophosphate pesticides. Therefore, there are common fragments as 62.96304 or 78.95795 *m/z*, which were observed for fosetyl aluminum, glyphosate, AMPA and N-acetyl-AMPA fragmentation (Table 1).

Additionally, spectrometric parameters were optimized, such as spray voltage 2.5, 3, 3.5 and 4 kV; capillary temperature 150, 200 and 300 °C and capillary voltage: −20, −25 and −35 V. The optimal parameters were 4 kV, 300 °C and −35 V, respectively.

### 3.2. Optimization of Chromatographic Separation

Orbitrap allows the identification of coeluting compounds with high accuracy based on the exact masses, but optimal chromatographic separation is necessary due to the presence of common fragments and an unequivocal identification of each compound is mandatory.

In this study, five different stationary phases (described in Section 2.2) were evaluated according to bibliography and a summary of the tested conditions are shown in Table S1 (see supplementary material). For optimization purposes, 10 µL of a mix solution of the compounds (1 mg L^−^^1^) were injected.

The first chromatographic column tested was Obelisc N, which was used for the analysis of polar pesticides. According to Botero-Coy et al. [15], water (0.1% formic acid) and acetonitrile were used as the mobile phase, and an isocratic mode proposed previously [16] with the aqueous phase (water 0.1% formic acid) and acetonitrile (20:80, *v/v*) was checked. First, the flow rate was set at 0.3 mL min^−1^ for 4.5 min, and then it was increased to 0.8 mL min^−1^ for 15 min. The temperature of the chromatographic column was 50 ºC. The obtained chromatogram is shown in Figure S1 (see supplementary material) and it can be observed that the signal of glyphosate was not sensitive and peak shape of AMPA was not acceptable, as well as high noise was observed for ethephon.

Then, the HILIC-A stationary phase was tested. The same mobile phase checked before was used (see Table S1). In this case, an elution gradient was tested, starting at 100% of acetonitrile, which was kept constant for 5 min, and then decreased to 60% for 1 min and it was held for 17 min before coming back to the initial conditions in 1 min. Finally, 20 min was used as post-equilibration time to allow the column to equilibrate prior to the next injection [27]. The flow rate was 0.3 mL min^−1^. According to the results shown in Figure S2, this column provided better peak shape and sensitivity, but an adequate separation of the analytes was not achieved, since some compounds as HEPA, glyphosate and N-acetyl-glyphosate coeluted. Other gradient profiles were tested (data not shown) but similar results were obtained.

Then, the HILIC-B stationary phase was evaluated as well as Zorbax HILIC Plus. The same mobile phases, elution gradient and post-equilibration time tested for HILIC-A, were used. Applying these conditions, no chromatographic peaks were obtained for the analytes of interest when HILIC-B was used, as these may have eluted without interaction with the active sites of the column. When the Zorbax HILIC Plus column was checked, chromatograms were obtained for the analytes but there was no chromatographic separation and broad peaks were observed for some of them (Figure S3). It can be observed that different results were obtained when different HILIC stationary phases were tested indicating that a different mechanism could be involved in the polar analyte partitioning [28].

Finally, the Torus DEA stationary phase from Waters was tested. It was developed for the separation of polar compounds, as those included in this study, and two elution gradients were tested. On the one hand, Method A comprises of (A) 50 mM ammonium formate aqueous solution (0.9% formic acid), and (B) acetonitrile (0.9% formic acid) as the mobile phase. The gradient elution started at 10% of A and increased to 60 % in 4.5 min. This composition was held for 11 min before coming back to initial conditions (10% A) in 1 min. Finally, this composition was held for 1 min. On the other hand, Method B uses (A) water (0.9% formic acid) and (B) acetonitrile (0.9% formic acid) as components of the mobile phase. The gradient elution started at 10 % of A and increased to 85 % A in 4 min. This composition was held for 14 min, before coming back to the initial conditions (10% A) in 1 min. Finally, this composition was held for 1 min. In both cases, column temperature was kept at 50 °C as it was recommended by Waters [29].

**Figure 2:**
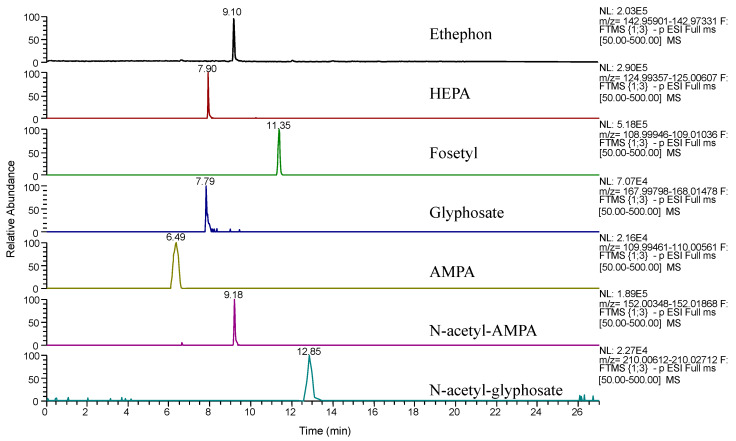
Extracted ion chromatograms of a standard solution of the targeted compounds (1000 µg L^−1^) using Torus DEA and the optimized chromatographic conditions described in Section 2.5.

When Method A was tested, an adequate chromatographic separation was not achieved for all the tested compounds (see Figure S4), although narrow peaks were obtained for most of them. When Method B was tested, the chromatographic separation was achieved for the targeted analytes (Figure S5) but glyphosate and N-acetyl-glyphosate show neither suitable sensitivity nor peak shape. In order to improve the elution of these two compounds, Method B was slightly modified, and the gradient profile described in Section 2.5 was used. As it can be observed in Figure 2, suitable peak shapes and elution of the target compounds were achieved. Additionally, retention times were reduced, and for instance, for ethephon, it was decreased from 12.06 to 9.10 min. Therefore, the Torus DEA stationary phase and chromatographic conditions described in Section 2.5 were used for further analysis.

### 3.3. Optimization of Extraction Method

The extraction method was optimized using the QuPPe procedure [7] as the starting point. This procedure was tested in the four selected matrices and suitable recoveries were achieved in tomato and grape (Table 2), whereas recoveries lower than 70% were obtained in orange and aubergine. However, it was observed that for aubergine and orange, the addition of water was needed to achieve suitable results. This was also observed by previous researchers [16], as well as it is recommended by EURL laboratory [7]. Thus, 1 mL should be added to aubergine and 1.5 mL of water to orange to minimize volumetric errors and make easier the homogenization step. 

Bearing in mind that some analytes, such as fosetyl-Al, are systemic pesticides, it is essential to quickly break down plant tissues. Therefore, the use of polytron was evaluated, and the four matrices were spiked with the targeted compounds at 250 µg kg^−1^ (three replicates) and polytron was used for 1 min, showing the results in Table 2. It can be observed that recoveries and repeatability were better when polytron was used, so it was applied for further experiments. Although recoveries for the targeted compounds were improved, they were still lower (39–71%) in aubergine, so n-acetyl-d_3_-glufosinate was used as the internal standard. Thus, for this matrix, 500 µg kg^−1^ was added to the sample before the extraction and it can be observed (see Table 2) that recoveries considerably improved for the target compounds (from 78% to 94%). Therefore, in addition to polytron, the use of an internal standard was needed for the analysis of these pesticides in aubergine, whereas in the other matrices, it was not necessary.

### 3.4. Method Validation

The optimized method for each matrix was validated for the target compounds using current SANTE Guidelines (SANTE 12682/2019) [26].

Firstly, linearity was evaluated throughout determination coefficients (R^2^) and they were >0.999 in all the cases. In addition, the standard deviation of the residuals was lower than 20%.

Regarding matrix effect (see Table 3), which was calculated using Equation (1), significant enhancement (positive) matrix effect (>20%) was observed for the tested compounds in tomato and grape matrices, whereas suppression (negative) effect (<−20%) was obtained in orange. Nevertheless, matrix effect was not significant (−20 < matrix effect < 20%) in aubergine because the addition of the internal standard. Therefore, matrix matched calibration was used for quantification of the target compounds in tomato, grape and orange, whereas in aubergine, the addition of the internal standard was also needed.

LOQs ranged between 25 and 100 µg kg^−1^, depending on the combination of compound/matrix evaluated. The highest values were obtained for AMPA, n-acetyl AMPA and n-acetyl glyphosate in orange and aubergine. Nevertheless, these values are equal to or lower than the MRLs set by the EU [30] for these matrices. For instance, the lowest MRL set by the EU for this type of compounds is 50 µg kg^−1^, which was established for ethephon in orange and aubergine. 

The average recoveries ranged between 70%–103% in tomato, 74–97% in grape, 74–105% in orange and 85–110% in aubergine (Table 3). Overall, recoveries were suitable for all matrices due to the fact that these values are between 70–110%.

Intra-day precision was always equal to or below 9.4% in tomato, 12.8% in grape, 15.4% in orange and 12.4% in aubergine, whereas inter-day precision was always below 19.0% for the tested compounds in the four matrices evaluated (Table 3). Although these values are slightly higher than those obtained by ion chromatography [19], they are lower than 20%, which is the maximum level set by SANTE guidelines [26] and similar to those obtained in previous studies [15].

### 3.5. Sample Analysis

The analytical method was applied to the analysis of the target compounds in 40 samples (10 samples from each matrix). In order to ensure the reliability of the results, an internal quality control was used. Thus, a reagent blank, a matrix blank, a spiked sample at the LOQ of the target compounds, and a matrix matched calibration were injected in every sequence of samples in order to check the stability of the proposed method.

**Figure 3:**
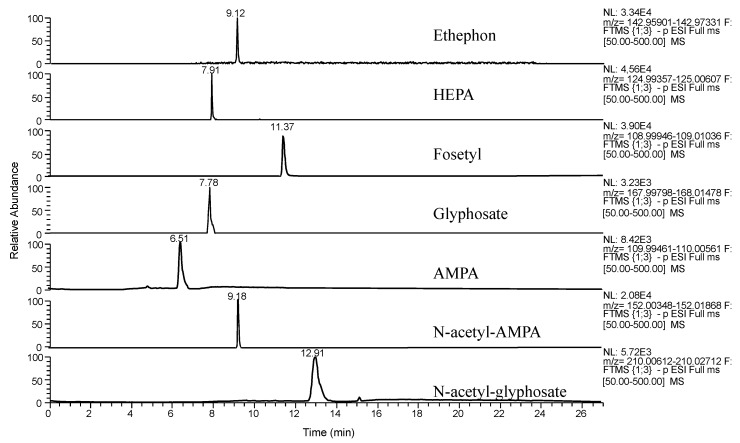
Extracted ion chromatograms from a spiked aubergine sample (100 µg kg^−1^) of the targeted compounds.

The compounds were not detected in the analyzed samples. Figure 3 shows the extracted ion chromatograms of a spiked aubergine sample at LOQ, and it can be observed that suitable peak shape was obtained for the target compounds and no interferences were detected.

Finally, and comparing the proposed methodology with previous methods, it must be highlighted that the derivatization procedure is not needed [6], simplifying sample handling. Additionally, similar sensitivity than that obtained by ion chromatography can be achieved [23] with a shorter running time, and a higher number of compounds can be analyzed simultaneously than using other conventional columns as Hypercarb [15].

## 4. Conclusions

A pluri-residue method was developed and validated for the simultaneous determination of polar pesticides in fruits and vegetables. After evaluation of different stationary phases, TORUS DEA column shows an adequate separation of the analytes. Despite the fact that QuPPe is a well-established procedure for the extraction of the targeted compounds from fruits and vegetables, several modifications, such as the use of polytron, were performed in order to improve the recovery of polar pesticides from different matrices. Additionally, it was noted that different amounts of water should be added to the sample depending on the type of matrix. For the detection of the compounds, high resolution single mass spectrometer, such as an Exactive-Orbitrap analyzer, provided a reliable identification, taking into account that in addition to the characteristic ion, at least two fragments were monitored per compound. Additionally, retrospective analysis can be performed in order to detect suspect compounds. The proposed method is an interesting alternative to previous methodologies, considering that shorter running times were achieved for the analyses of a higher number of compounds at concentrations equal to or below the MRL set by EU.

## Figures and Tables

**Table 1:** HRMS parameters for the polar pesticides.

Analyte	Characteristic Ion		Fragment Ion		
	Exact Mass	Mass Error (ppm)	Exact Mass	Molecular Formule	Mass Error (ppm)
*Ethephon*	142.96616	1.576	78.95795	[O_3_P]^−^	−0.087
			106.98926	[C_2_H_6_O_4_P]^−^	−0.533
*HEPA*	124.99982	1.827	59.01276	[C_2_H_3_O_2_]^−^	2.611
			78.95795	[O_3_P]^−^	0.293
			94.98926	[CH_4_O_3_P]^−^	−1.232
*Fosetyl-Al*	109.00491	0.027	62.96304	[O_2_P]^−^	0.281
			78.95795	[O_3_P]^−^	−0.087
			80.97361	[H_2_PO_3_]^−^	0.161
*Glyphosate*	168.00673	3.243	62.96304	[O_2_P]^−^	−1.307
			78.95795	[O_3_P]^−^	−2.367
			80.97361	[H_2_PO_3_]^−^	3.360
			124.01581	[C_2_H_7_O_3_NP]^−^	−2.065
*AMPA*	110.00125	−0.419	62.96304	[O_2_P]^−^	0.281
			78.95795	[O_3_P]^−^	−0.721
*N-acetyl-AMPA*	152.01182	2.364	62.96304	[O_2_P]^−^	1.393
			78.95795	[O_3_P]^−^	0.926
*N-acetyl-glyphosate*	210.01730	2.000	62.96304	[O_2_P]^−^	1.711
			124.01581	[C_2_H_7_O_3_NP]^−^	−0.936
			148.01581	[C_4_H_7_O_3_NP]^−^	3.329
*N-acetyl-d3-glufosinate (ILIS)*	225.07196	1.832			

**Table 2:** Recovery values obtained after the application of several extraction procedures.

Compound	Matrix	QuPPe	Water Addition ^a^	Polytron	Internal Standard ^b^
*Ethephon*	Tomato	119 (6)^c^		111 (2)	
	Grape	83 (12)		85 (7)	
	Orange	43 (13)	82 (6)	100 (4)	
	Aubergine	<30	<30	42 (14)	78 (10)
*HEPA*	Tomato	88 (8)		113 (2)	
	Grape	58 (20)		72 (14)	
	Orange	68 (14)	82 (5)	105 (5)	
	Aubergine	<30	<30	56 (23)	82 (12)
*Fosetyl-Al*	Tomato	92 (7)		90 (1)	
	Grape	70 (18)		79 (10)	
	Orange	58 (18)	79 (5)	95 (4)	
	Aubergine	<30	<30	56 (20)	78 (13)
*Glyphosate*	Tomato	90 (12)		92 (8)	
	Grape	80 (13)		84 (5)	
	Orange	65 (13)	75 (12)	73 (8)	
	Aubergine	<30	<30	39 (15)	85 (12)
*AMPA*	Tomato	73 (15)		76 (10)	
	Grape	71 (9)		73 (4)	
	Orange	45 (20)	84 (18)	89 (5)	
	Aubergine	<30	<30	41 (20)	79 (9)
*N-acetyl-AMPA*	Tomato	92 (17)		96 (11)	
	Grape	89 (6)		91 (4)	
	Orange	65 (12)	81 (23)	79 (11)	
	Aubergine	<30	<30	51 (23)	94 (8)
*N-acetyl-glyphosate*	Tomato	82 (14)		84 (11)	
	Grape	72 (9)		75 (6)	
	Orange	45 (29)	65 (25)	72 (13)	
	Aubergine	<30	<30	52 (19)	80 (10)

^a^ Evaluated only in orange (1.5 mL) and aubergine (1 mL); ^b^ Evaluated only in aubergine using n-acetyl-d3-glufosinate; ^c^ Relative standard deviation in brackets (*n* = 3).

**Table 3:** Validation results.

Matrix	Compound	Matrix Effect	LOQ (µg kg^−1^)	Recovery (%) ^a^	Precision ^b^
Tomato	Ethephon	46	25	100–70	6.7 (9.5)
	HEPA	51	25	94–97	5.2 (10.9)
	Fosetyl-Al	37	25	78–70	8.9 (9.9)
	Glyphosate	38	25	73–85	7.4 (15.6)
	AMPA	36	50	82–98	8.6 (11.3)
	N-acetyl-AMPA	20	50	103–75	9.4 (10.8)
	N-acetyl-glyphosate	34	50	98–91	5.8 (13.5)
Grape	Ethephon	36	25	97–79	7.0 (13.9)
	HEPA	44	25	82–90	8.9 (15.3)
	Fosetyl-Al	31	25	76–87	7.8 (9.7)
	Glyphosate	75	25	74–83	12.8 (15.2)
	AMPA	69	50	86–81	4.6 (8.4)
	N-acetyl-AMPA	48	50	94–89	8.2 (11.3)
	N-acetyl-glyphosate	−86	50	81–83	12.4 (15.4)
Orange	Ethephon	−33	50	77–102	11.6 (15.8)
	HEPA	−21	25	86–105	15.4 (18.3)
	Fosetyl-Al	−27	25	91–88	9.9 (11.3)
	Glyphosate	−26	50	102–79	9.9 (14.4)
	AMPA	−40	100	90–74	6.7 (10.8)
	N-acetyl-AMPA	−31	100	77–80	7.2 (10.3)
	N-acetyl-glyphosate	−43	100	86–88	7.8 (10.9)
Aubergine	Ethephon	18	50	92–108	9.7 (13.5)
	HEPA	14	25	97–86	9.6 (15.3)
	Fosetyl-Al	21	25	90–105	8.4 (13.5)
	Glyphosate	25	50	95–85	12.4 (15.2)
	AMPA	14	100	102–91	5.6 (12.0)
	N-acetyl-AMPA	18	100	110–104	9.1 (16.3)
	N-acetyl-glyphosate	17	100	93–100	4.6 (8.4)

^a^ Recovery values at LOQ and 10 times LOQ.; ^b^ Intraday precision at LOQ. Inter-day precision at LOQ is given in parenthesis. In both cases, n = 5.

## Supplementary Materials

The following are available online at https://www.mdpi.com/2304-8158/9/5/553/s1, Table S1. Chromatographic conditions tested during the optimization of the LC method; Figure S1: Extracted ion chromatograms of a standard solution of the targeted compounds (1000 µg L^−1^) using Obelisc N as stationary phase; Figure S2: Extracted ion chromatograms of a standard solution of the targeted compounds (1000 µg L^−1^) using HILIC-A as stationary phase; Figure S3: Extracted ion chromatograms of a standard solution of the targeted compounds (1000 µg L^−1^) using Zorbax HILIC Plus as stationary phase; Figure S4: Extracted ion chromatograms of a standard solution of the targeted compounds (1000 µg L^−1^) using TORUS DEA as stationary phase and applying method A as gradient profile; Figure S5: Extracted ion chromatograms of a standard solution of the targeted compounds (1000 µg L^−1^) using TORUS DEA as stationary phase and applying method B as gradient profile.
