# Supplementary material for: Development and Application of a Novel Pluri-Residue Method to Determine Polar Pesticides in Fruits and Vegetables through Liquid Chromatography High Resolution Mass Spectrometry
Source: Foods. 2020 May 1;9(5):553. doi: 10.3390/foods9050553 (PMC7278595; doi:10.3390/foods9050553)
Supplement: Supplementary file 1 [file foods-09-00553-s001.zip › foods-770073-supplementary.docx]

Journal name: Foods

SUPPLEMENTARY MATERIAL to the paper entitled: Development and application of a novel pluri-residue method to determine polar pesticides in fruits and vegetables through liquid chromatography-high resolution mass spectrometry

Lorena Manzano Sánchez, José Antonio Martínez Martínez, Irene Domínguez, José Luis Martínez Vidal, Antonia Garrido Frenich, Roberto Romero-González*

Department of Chemistry and Physics, Analytical Chemistry Area, University of Almeria, Center for Research in Mediterranean Intensive Agrosystems and Agri-Food Biotechnology (CIAIMBITAL), Agrifood Campus of International Excellence ceiA3, Carretera de Sacramento s/n, E-04120 Almeria, Spain

*****Correspondence: rromero@ual.es; Tel.: +34950214278

E-mail address: rromero@ual.es (R. Romero-González).

**Table of contents**

**Table S1.** Chromatographic conditions tested during the optimization of the LC method.

**Figure S1**. Extracted ion chromatograms of a standard solution of the targeted compounds (1000 µg L^-1^) using Obelisc N as stationary phase.

**Figure S2.** Extracted ion chromatograms of a standard solution of the targeted compounds (1000 µg L^-1^) using HILIC-A as stationary phase.

**Figure S3.** Extracted ion chromatograms of a standard solution of the targeted compounds (1000 µg L^-1^) using Zorbax HILIC-Plus as stationary phase.

**Figure S4.** Extracted ion chromatograms of a standard solution of the targeted compounds (1000 µg L^-1^) using TORUS DEA as stationary phase and applying method A as gradient profile.

**Figure S5.** Extracted ion chromatograms of a standard solution of the targeted compounds (1000 µg L^-1^) using TORUS DEA as stationary phase and applying method B as gradient profile.

**Table S1.** Chromatographic conditions tested during the optimization of the LC method

| **Stationary phase** | **Mobile phase** | **Flow rate** | **Elution mode** |
| --- | --- | --- | --- |
| Obelisc N | A: Water (0.1% formic acid)  B: Acetonitrile | 0.3 mL min^-1^ (4.5 min)  0.8 mL min^-1^ (15 min) | Isocratic mode (20 A:80 B, *v/v*) |
| HILIC-A | A: Water (0.1% formic acid)  B: Acetonitrile | 0.3 mL min^-1^ | Gradient mode: 100 % B (5 min) → 60 % B (in 1 min) → 60 % B (constant 16 min) → 100 % B (1 min) → Post-equilibration time (20 min) |
| HILIC-B |  |  |  |
| Zorbax-HILIC Plus |  |  |  |
| Torus DEA (Method A) | A: Water (50 mM ammonium formate & 0.9 % formic acid)  B: Acetonitrile (0.9 % formic acid) | 0.5 mL min^-1^ | Gradient mode: 10 % A → 60 % A (1 min) → 60 % A (4.5 min) → 60 % A (11 min) → 10 % A (1 min) |
| Torus DEA (Method B) | A: Water (0.9 % formic acid)  B: Acetonitrile (0.9 % formic acid) | 0.5 mL min^-1^ | Gradient mode: 10 % A → 85 % A (4 min) → 85 % A (14 min) → 10 % A (1 min) |
| Torus DEA (Modified Method B)^1^ | A: Water (0.9 % formic acid)  B: Acetonitrile (0.9 % formic acid) | 0.5 mL min^-1^ | Gradient mode: 10 % A → 90 % A (6 min) → 90 % A (16 min) → 50 % A (2 min) → 10 % A (2 min) |

^1^ Selected chromatographic method for further experiments

**Figure S1**. Extracted ion chromatograms of a standard solution of the targeted compounds (1000 µg L^-1^) using Obelisc N as stationary phase.

**Figure S2.** Extracted ion chromatograms of a standard solution of the targeted compounds (1000 µg L^-1^) using HILIC-A as stationary phase.

**Figure S3.** Extracted ion chromatograms of a standard solution of the targeted compounds (1000 µg L^-1^) using Zorbax HILIC-Plus as stationary phase.

**Figure S4.** Extracted ion chromatograms of a standard solution of the targeted compounds (1000 µg L^-1^) using TORUS DEA as stationary phase and applying method A as gradient profile.

**Figure S5.** Extracted ion chromatograms of a standard solution of the targeted compounds (1000 µg L^-1^) using TORUS DEA as stationary phase and applying method B as gradient profile.
